# Novel mutation in *LRP5* gene cause rare osteosclerosis: cases studies and literature review

**DOI:** 10.1007/s00438-023-02008-2

**Published:** 2023-03-27

**Authors:** Dichen Zhao, Lei Sun, Wenbin Zheng, Jing Hu, Bingna Zhou, Ou Wang, Yan Jiang, Weibo Xia, Xiaoping Xing, Mei Li

**Affiliations:** Department of Endocrinology, National Health Commission Key Laboratory of Endocrinology, Peking Union Medical College Hospital, Chinese Academy of Medical Sciences and Peking Union Medical College, Shuaifuyuan No. 1, Dongcheng District, Beijing, 100730 China

**Keywords:** Low density lipoprotein receptor-related protein 5 (LRP5), Gene mutation, Autosomal dominant osteosclerosis type Ι (ADO Ι)

## Abstract

To study the effects of low-density lipoprotein receptor-related protein 5 (*LRP5*) gene mutations on bone, and to open up our view of LRP5 and Wnt pathways on bone mass regulation. Three patients with increased bone mineral density or thickened bone cortex were included, who were 30-year-old, 22-year-old and 50-year-old men, respectively. The latter two patients were son and father of a same family. The characteristics of bone X-rays were evaluated in detail. Bone turnover markers were detected, such as procollagen type 1 amino-terminal peptide (P1NP), alkaline phosphatase (ALP), and type 1 collagen carboxyl terminal peptide (β-CTX). Dual energy X-ray absorptiometry (DXA) was used to measure the bone mineral density (BMD) at lumbar spine and proximal femur of the patients. The targeted next-generation sequencing (NGS) technology was used to detect pathogenic gene mutations, which were further verified by Sanger sequencing. Moreover, the gene mutation spectrum and phenotypic characteristics of reported patients with *LRP5* gain-of-function mutations were summarized by reviewing the literature. The main characteristics of the first patient were headache, facial paralysis, high BMD (lumbar vertebrae 1–4: 1.877 g/cm^2^, Z-score: 5.8; total hip: 1.705 g/cm^2^, Z-score: 5.7), slightly increased P1NP (87.0 ng/mL) and β-CTX (0.761 ng/mL) level, and with thickened bone cortex, especially the cranial vault. The latter two patients showed enlargement of the mandible and enlarged osseous prominence of the tours palatinus. X-rays showed that the bone cortex of skull and long bones were thickened. The bone turnover markers and BMD were normal. All three cases carried novel missense mutations in *LRP5* gene, which were mutation in exon 3 (c.586 T > G, p.Trp196Gly) of the first patient, and mutation in exon 20 (c.4240C > A, p.Arg1414Ser) of the latter two patients. Combined with the reported literature, a total of 19 gain-of-function mutations in *LRP5* were detected in 113 patients from 33 families. Hotspot mutations included c.724G > A, c.512G > T and c.758C > T. Furthermore, mutations in the exon 3 of *LRP5* may cause severe phenotypes. *LRP5* gain-of-function mutations can lead to rare autosomal dominant osteosclerosis type Ι (ADO Ι), which was characterized by increased bone mass and thickened bone cortex. In-depth research on the Wnt pathway will be benefit for discovering important mechanisms of bone mass regulation.

## Introduction

The Wnt pathway is an important signaling pathway that widely exists and regulates various functions in the body, playing major roles in bone formation, growth and development (Clevers and Nusse 2012). Low density lipoprotein receptor-related protein 5 (LRP5) is one of the co-receptors of Wnt pathway, and its coding gene locates on chromosome 11q13 (Twells et al. 2001; He et al. 2004). Studies have shown that inactivating mutations of *LRP5* gene can decrease the activity of osteoblasts, resulting in osteoporosis pseudoglioma syndrome (OPPG), an autosomal recessive disorder characterized by extremely low bone mass (Joiner et al. 2013). However, activating mutations of *LRP5* gene can reduce the inhibitory effects of Dickkopf-1 protein (DKK-1) and sclerostin (SOST) on Wnt pathway, which could increase the activity of osteoblasts, leading to autosomal dominant osteosclerosis type I (ADO I), manifested by high-bone-mass (HBM) (Holdsworth et al. 2012; Balemans et al. 2008). In-depth research on the Wnt signaling pathway is helpful to understand the regulation mechanism of osteoblast function, and is of great significance for the development of drugs to promote bone formation, which have important prospects for the treatment of osteoporosis (Williams 2017).

Currently, ADO I caused by activating mutations of *LRP5* are reported as sporadic cases, which are rarely reported in the Chinese population. LRP5 protein is composed of an extracellular domain, a transmembrane domain, and a cytoplasmic domain. It is still unclear that if the effects of *LRP5* gene mutation on different receptor domains are related to the severity of the disease. Herein, this study investigated the effects of *LRP5* gene mutation on bone morphology, bone mineral density (BMD) and bone turnover biomarkers, and summarized the phenotypic characteristics of patients with gain-of-function mutations of *LRP5* gene by reviewing the literature. Also, we tried to analyze whether there was a correlation between disease genotype and phenotype in this study, to deepen the understanding of the role of LRP5 and Wnt signaling pathways in regulation of bone metabolism.

## Methods

### Subjects

**Fig. 1:**
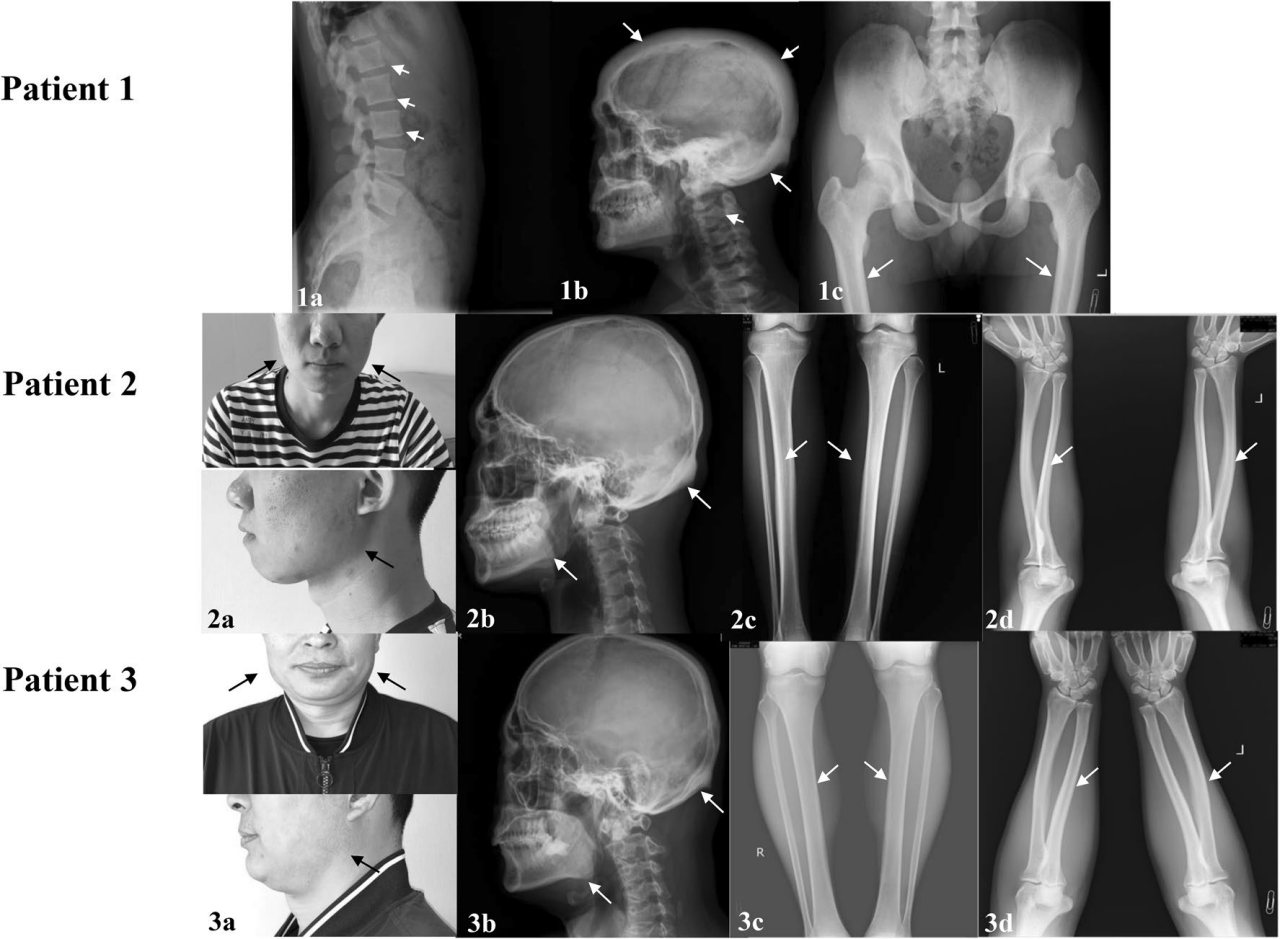
The physical findings and radiographic changes of patients in this study. (1**a**–**c**) The X ray films of lumber spine, cranial vault and hip of patient 1. The thickened bone cortex, narrowed bone marrow cavity of long bones. (2**a**、3**a**) The positive signs of patient 2 and 3. Mandibular enlargement and gonial angle protrusion. (2**b**–**d**) The X-ray films of long bones and skull of patient 2. A symmetrical cortical thickening of long bones, the skull with a normal external shape. An enlarged mandible could be seen in the X-ray of skull. (3**b**–d) The X-ray films of long bones and skull of patient 3. The thickening endosteum of the radius, ulna and the skull. A thickening bone cortex of the femur and fibula, and an enlargement of the mandible. The abnormal findings were indicated by *arrow*

**Fig. 2:**
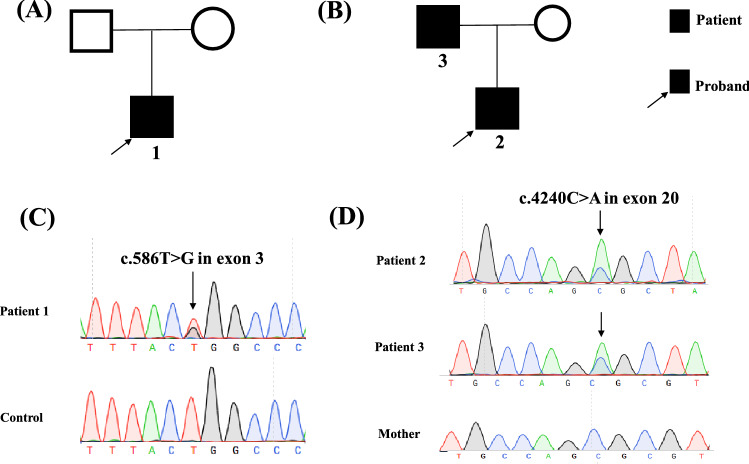
Pedigrees and Sanger sequencing results of patients in this study. **A** The pedigree of patient 1. **B** The pedigree of patient 2 and 3. The patients were son and his father. **C** A novel missense mutation in *LRP5* was identified in patient 1 as c.586 T > G in exon 3. **D** Another novel missense mutation in *LRP5* was identified in patient 2 and 3 as c.4240C > A in exon 20. Mutations were designated with *arrows*

Three patients were included in this study. Patient 1, 30 years old man, had facial muscle dyskinesia for more than 20 years, as well as headache for more than 10 years, and he visited the Endocrinology Department of Peking Union Medical College Hospital (PUMCH) in December 2018. At more than 10 years old, he presented with difficulty in closing left eye and baring teeth, and he was diagnosed as facial nerve palsy by the neurology department. Headache began to appear when he was more than 20 years old, and X-rays showed prominently thickened skull (Fig. 1). The patient had no vision or hearing loss, no history of fracture, and there was no family history of the similar disease. Physical examinations revealed height 175 cm, weight 68 kg, restriction in closing of left eyelid and showing teeth, protruding left skull, no obvious abnormalities in the remaining bones. Thyroid and cardiopulmonary examinations, muscle force and strength were normal. Patients 2 and 3 were a son and his father. Patient 2 came to the clinic of PUMCH in July 2015 due to facial skin swelling for 1 year. Since the age of 17, the patient developed facial skin thickening and stiffness, accompanied by protruding mandibular angles. He denied the remaining bone changes, bone pain, hearing or vision loss, or history of fracture. Physical examinations revealed height 175 cm, weight 60 kg, free movement, mandibular enlargement, protruding mandibular angles, prominent torus palatinus on the hard palate (Fig. 1), no obvious abnormalities in thyroid, cardiopulmonary and nervous system. Patient 3 had low back pain without other discomforts. Physical examinations showed hypertrophy of the mandible, enlarged gonial angles, and prominent torus palatinus on the hard palate. Three patients were followed up regularly for 2–5 years without special treatments. There was no significant change in the condition. Pedigrees of the patients were shown in Fig. 2.

100 healthy adults were included as the normal controls, whose sequenced data were collected as in-house database to determine whether novel mutations occurred as polymorphisms. This study was approved by the Scientific Research Ethics Committee of PUMCH, and all participants signed the informed consents.

## Evaluation of the phenotypes

Medical history was collected in detail, including bone pain, nerve compression, fractures and skeletal deformities. Detailed physical examinations were performed, including bones, teeth, nerves and motor systems. Skull, thoracolumbar spine, long bones and pelvis were examined by X-ray. Dual energy X-ray absorptiometry (DXA, GE Lunar Prodigy) was used to measure the areal BMD at lumbar spines 1–4 (L1-4), femoral neck, trochanter, and total hip. The Z-scores were calculated by comparing the measured BMD with the mean BMD values obtained in the normal population of the same age and sex. The reference range of the normal population referred to the examination results of 475 healthy volunteers of different ages in PUMCH (Yu et al. 1996). Fasting venous blood and urine samples of research subjects were collected for measurements of blood calcium, phosphorus, alkaline phosphatase (ALP, bone formation marker), alanine aminotransferase (ALT), creatinine (Cr), 24-h urine calcium and urine phosphorus levels using an automatic biochemical analyzer. Serum type 1 collagen carboxyl terminal peptide (β-CTX, a bone resorption marker), procollagen type 1 amino-terminal peptide (P1NP, a bone formation marker), 25-hydroxy-vitamin D (25OHD) and parathyroid hormone (PTH) levels were detected using an automated electrochemiluminescence system (Roche Diagnostics, Switzerland) of the clinical central laboratory of PUMCH.

## Detection of the pathogenic gene mutation

**Fig. 3:**
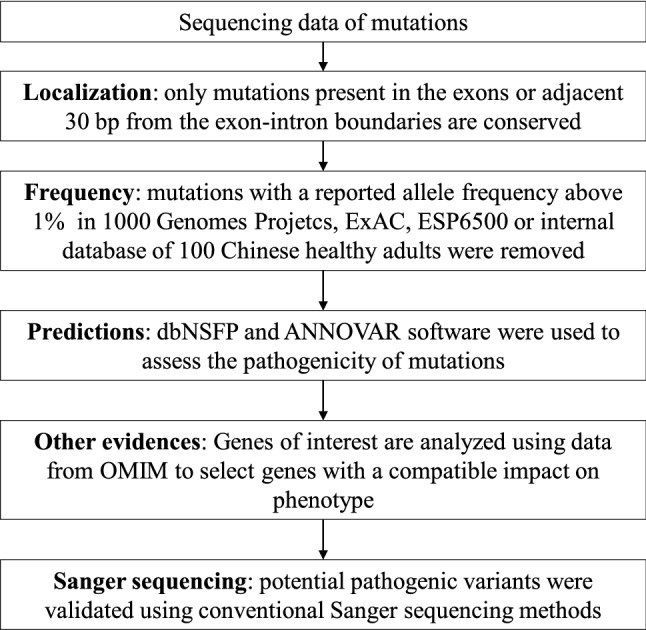
Flowchart of data analysis to identify disease-causing mutations

Genomic DNA of peripheral blood leukocyte of patients, their family members and 100 unrelated healthy subjects were extracted using the DNA Extraction kit (QIAamp DNA; Qiagen, Germany). Sequence of the parents of patient 1 was unavailable in this study, because the proband declared that their parents had no similar symptoms, and they were unwilling to receive genetic sequencing. DNA samples of the patients were sequenced by a targeted next-generation sequencing (NGS) panel (Illumina HiSeq2000 platform, Illumina, Inc., San Diego, CA, USA). As previously described (Liu, et al. 2017), the capture panel of targeted DNA covered 14 known candidate genes of osteogenesis imperfecta (OI), and 708 other genes related to other bone disorders, including congenital rickets, cartilage diseases, and osteosclerosis. Overall, the targeted panel included a total of 722 genes, which compromised 9712 exons. The sequencing was performed with a coverage rate of 98.95% and an average depth of 200 × on target regions in each of the chromosomes (Liu, et al. 2017). SOAP SNP software was used to remove SNPs. The annotation and filtering procedure are as following: Insertions or deletions were analyzed using BWA (http://bio-bwa.sourceforge.net/) and GATK programs (https://www.broadinstitute.org/gatk/). Variants with allele frequency more than 1% in ESP6500, ExAC, 1000 Genomes Project, ChinaMAP, WBBC as well as the in-house sequencing data of 100 Chinese Han normal controls were filtered. To predict the effect of missense variants, we used dbNSFP and ANNOVAR, which contains seven well-established in silico prediction programs (Scale-Invariant Feature Transform [SIFT], Polyphen2, LRT, MutationTaster, and PhyloP). Uniprot software was used to analyze the conservation of the amino acid substitution among different species. The data analysis was performed following the pipelines previously described (Liu, et al. 2017; Asan, et al. 2017), including annotation at the gene levels and variant levels (Fig. 3). Variants were prioritized and interpreted following a criterion modified from the American College of Medical Genetics and Genomics (ACMG) recommended standards (Riggs et al. 2020).

Polymerase chain reaction (PCR) and Sanger sequencing were used to further verify pathogenic gene mutations. Primer3 (http: //bioinfo.ut.ee/primer3-0.4 0.0/) was used to design primers. TaqDNA kit (Biomed, China) was used to amplify the target gene exon and its flanking sequence. For patient 1, the exon 3 of *LRP5* gene was amplified with the primer sequence: 5′-TGGTTATTTCCGATGGGTGAGA-3′, 5'-GACGCTGTTCCAAGTTCTGAG-3′. For patients 2, 3 and their family member, exon 20 of *LRP5* gene was amplified with the primer sequence: 5'-ATGTTGGCCACCTCTTTCTG-3', 5'-CTGCCTCCTCCAGATCATTC-3'. Also, exon 3 and 20 of *LRP5* gene of 100 healthy subjects were amplified to exclude the possibility of gene polymorphism. PCR reaction conditions: initial denaturation at 95 °C for 3 min, followed by 35 cycles of denaturation at 95 °C for 30 s, annealing at 57–61 °C for 30 s, extension at 72 °C for 1 min, and a final extension at 72ºC for 15 min. After the PCR amplification products were purified, they were directly sequenced using an ABI377 DNA automated sequencer with dye terminator cycle sequencing kits (Applied Biosystems), and the results were compared with the reference sequence NM_002335.4. to determine the mutation site and type.

## Literature review

“*LRP5* mutation”, “high bone mass”, “osteopetrosis/osteosclerosis”, “ADO I” were used as key words to search for foreign literatures in the PubMed database. Key words were also searched in the China National Knowledge Infrastructure (CNKI) database and Wanfang database for Chinese literatures. The characteristics of pathogenic genotypes and disease phenotypes of patients with osteosclerosis caused by *LRP5* gain-of-function mutations were summarized, and to analyze whether there was a correlation between the genotypes and phenotypes.

## Results

### Clinical phenotypes

The clinical phenotypes of patient 1 included headache, facial paralysis, and thickened skull. The ALP level was normal (121U/L), β-CTX (0.761 ng/mL) and P1NP (87.0 ng/mL) levels were slightly elevated. The vitamin D was severely deficient (25OHD 9.3 ng/mL) with secondary hyperparathyroidism (107.2 pg/ml). Besides, the BMD was significantly increased as L1-4 BMD value 1.877 g/cm^2^ (Z-score 5.8), femoral neck 1.675 g/cm^2^ (Z-score 5.9), trochanter 1.276 g/cm^2^ (Z-score 4.1), total hip 1.705 g/cm^2^ (Z-score 5.7) (Table 1). X-rays showed thickened bone cortex in the whole body, narrowed bone marrow cavity, and thickened cranial vault (Fig. 1).

**Table 1:** Clinical characteristics of patients in this study

	Patient 1	Patient 2	Patient 3	Reference range
Age(years)	30	22	50	/
Gender	M	M	M	
Height(cm)	175	175	172	/
Weight(kg)	68	60	80	/
Symptoms	Headache, left facial nerve palsy	Swelling of face	Low back pain	/
The enlargement of mandible	No	Yes	Yes	/
The prominence of torus palatinus	No	Yes	Yes	/
ALT(U/L)	20	12	17	9–50
Cr(μmol/l)	71	78	70	59–104
Ca(mmol/L)	2.33	2.48	2.27	2.13–2.70
P(mmol/L)	1.03	1.43	1.36	0.81–1.45
PTH(pg/mL)	*107.2*	44.1	55.5	12.0–68.0
25OHD(ng/mL)	*9.3*	*16.9*	*13.9*	20.0–50.0
ALP(U/L)	121	67	67	45–125
β-CTX(ng/mL)	*0.761*	0.43	0.39	0.26–0.512
P1NP(ng/mL)	*87.0*	60.9	41.9	15.1–58.6
LS BMD(g/cm^2^)	1.877	1.216	1.109	Yu et al. (1996)
LS BMD Z-score^*^	*5.8*	0.5	-0.5	/
FN BMD(g/cm^2^)	1.675	1.082	1.034	Yu et al. (1996)
FN BMD Z-score^*^	*5.9*	0.7	1.0	/
TR BMD(g/cm^2^)	1.276	0.927	0.796	Yu et al. (1996)
TR BMD Z-score^*^	*4.1*	0.9	0.0	/
TH BMD(g/cm^2^)	1.705	1.152	1.038	Yu et al. (1996)
TH BMD Z-score^*^	*5.7*	1.3	0.7	/
Radiographic manifestations	Diffusely thickening of the bone cortex, especially the cranial vault	Enlarged mandible, and thickened cortex of the long bones	Thickening of the cortical bones in tibia and femur, enlargement of the mandible	/

Abnormal results were indicated in *italic*

*ALT* alanine aminotransferase, *Cr*: creatinine, *PTH*: parathyroid hormone; 25OHD: 25 hydroxyvitamin D, *ALP*: alkaline phosphatase; *β-CTX*: type 1 collagen carboxyl terminal peptide, *P1NP*: procollagen type 1 amino-terminal peptide; *BMD*: bone mineral density, *LS*: lumbar spine 1–4, *FN*: femoral neck; *TR*: trochanter, *TH*: total hip

* A Z-score was calculated by comparing the measured BMD with the mean BMD values obtained in a population of the same age and gender

Yu et al. 1996) The reference 8

Patients 2 and 3 had characteristic facial changes, including prominent torus palatinus, mandibular enlargement, and gonial angles protrusion (Fig. 1). Patient 2 presented with facial skin thickening without bone pain or nerve compression. Two patients had vitamin D deficiency (25OHD levels were 16.9 and 13.9 ng/mL, respectively), normal P1NP, β-CTX and ALP levels. Radiographic films showed enlarged mandible, and thickened bone cortex of long bones (Fig. 1). DXA results were normal (Table 1).

## Pathogenic gene mutation in *LRP5*

Two missense mutations of *LRP5* were detected in this study. The mutation of patient 1 was c.586 T > G in the exon 3 of *LRP5*, which caused p.Trp196Gly. That is, the 196^th^ amino acid was changed from tryptophan to glycine. Patient 2 and 3 had c.4240C > A mutation in exon 20 of *LRP5*, which caused p.Arg1414Ser. That is, the 1414^th^ amino acid was changed from arginine to serine. The allele frequency of the two sites of *LRP5* (c.586; c.4240) in Asian people is 0.000972 and 0.000092 according to ExAC. The mutations mentioned above were not detected in other family members of the patients, as well as in 100 unrelated healthy subjects. By searching the published papers in HGMD, LOVD3.0 database and PubMed, it was confirmed that the *LRP5* mutations detected in this study were firstly reported. Meanwhile, according to the predictions of dbNSFP and ANNOVAR software, the two mutations were all pathogenic. The alignment of homologous sequences showed that the affected amino acids were highly conserved among different species.

## Literature review and analysis

By summarizing the literature, there was a total of 113 patients from 33 families in 12 countries carrying *LRP5* gene activating mutations, of which 54 cases were men (47.8%) and 59 cases were women (52.2%), and these patients all showed normal growth and development, intelligence, and life span (Wesenbeeck et al. 2003). The patients were mostly discovered accidentally or detected after the identification of the proband in the same family, with the average age at diagnosis was 43 years old. The clinical phenotypes mainly included: mandibular enlargement (51/57), prominence of torus palatinus (44/62), bone pain (21/59), headache (12/51), hearing loss (9/54), facial nerve palsy (7/56), and vision loss (2/53). The clinical manifestations were heterogeneous, and the mild cases could present with no subjective symptoms (Renton et al. 2002). The radiographic changes were mainly the thickened bone cortex of the skull and long bones (Huybrechts et al. 2020; Beals et al. 2001), and increased BMD (the proportion of patients with elevated BMD at lumbar spine and hip were 40/43, 26/31, respectively), with an average BMD Z-score of 6.2 ± 2.5 and 5.1 ± 3.1 at lumbar spine and total hip, respectively (Table 2) (Wesenbeeck et al. 2003; Renton et al. 2002; Beals et al. 2001; Gregson et al. 2016; Yuan et al. 2020; Janer Subías et al. 2015; Rickels et al. 2005; Boyden et al. 2002; Whyte et al. 2004; Little et al. 2002; Roetzer et al. 2018; Pangrazio et al. 2011; Costantini et al. 2017; Kwee et al. 2005; Wang et al. 2013; Frost et al. 2003; Balemans et al. 2007). Most patients with *LRP5* activating mutations had normal ALP (15/17), P1NP (3/6) and β-CTX (10/15) levels. Compared with control group with normal bone mass, there was no significant difference in the levels of bone formation markers: ALP and P1NP, while the bone resorption biomarker β-CTX was decreased or normal (Frost et al. 2003; Hernandez-Cassis et al. 2003). ADO I is in autosomal dominant inheritance. 19 gain-of-function mutations of *LRP5* were found so far, the main mutation type was the missense mutation (17/19), and one insertion mutation (c.509_514dupGGGGTG) and one deletion mutation (c.511_516delGGTGAG) were also found (Table 3). The high-frequency mutation sites were c.724G > A (6/33), c.512G > T (5/33) and c.758C > T (4/33), and there was no obvious discrepancy in ethnic distribution, and mutations located in exon 3 were more likely to cause the severe phenotype.

**Table 2:** The clinical characteristics of reported patients with ADO I in literature and in this study

	Clinical characteristics (proportion)^*^
Number of cases	113
Mean age at diagnosis (y) (range)	43 (1–83)
Gender	M:F = 54:59
Headache	23.5% (12/51)
Facial nerve palsy	12.5% (7/56)
Hearing loss	16.7% (9/54)
Vision loss	3.8% (2/53)
Bone pain	35.6% (21/59)
Torus palatinus prominence	71.0% (44/62)
Mandible enlargement	89.5% (51/57)
Normal serum ALP levels	88.2% (15/17)
Normal serum P1NP levels	50.0% (3/6)
Normal serum β-CTX levels	66.7% (10/15)
LS BMD Z-score > 2.5SD	93.0% (40/43)
LS BMD Z-score (mean ± SD)	6.2 ± 2.5
TH BMD Z-score > 2.5SD	84.0% (26/31)
TH BMD Z-score (mean ± SD)	5.1 ± 3.1

*ALP* alkaline phosphatase, *β-CTX* type 1 collagen carboxyl terminal peptide, *P1NP* procollagen type 1 amino-terminal peptide, *BMD* bone mineral density, *LS* lumbar spine 1–4, *FN* femoral neck, *TH* total hip

*Percentage represents the proportion of patients with the listed clinical feature over total reported cases

**Table 3:** Reported genotypes and phenotypes of *LRP5* activating mutations

	Mutation position	Mutation site	Amino acid change	Family numbers (ethnicity)	Number of patients with severe phenotype (proportion)^a^	Mean Z-score of BMD		Reference
						LS	TH	
1	Exon 2	c.266A > G	Q89R	1 (1British)	0 (0/1)	4.6	2.4	14
2	Exon 2	c.331G > T	D111Y	2 (1Argetinean, 1Chinese)	0 (0/6)	6.3	7.2	10,15
3	Exon 2	c.335G > T	G112V	1 (1Spanish)	1 (1/2)	5.1	/	16
4	Exon 2	c.461G > T	R154M	1 (1American)	0 (0/6)	8.0	8.7	17
5	Exon 3	c.511G > C	G171R	1 (1Belgian)	0 (0/3)	/	/	10
6	Exon 3	c.512G > T	G171V	5 (5American)	5 (5/36)	6.7	5.4	18–20
7	Exon 3	c.509_514dupGGGGTG	G171_E172insGG	1 (1Australian)	1 (1/2)	8.4^*^	9.6^*^	21
8	Exon 3	c.511_516delGGTGAG	G171_E172del	1 (1Italian)	1 (1/1)	/	/	22
9	Exon 3	c.518C > T	T173M	1 (1British)	0 (0/1)	4.2	3.6	14
10	Exon 3	c.586 T > G	W196G	1 (1Chinese)	1 (1/1)	5.8	5.7	This study
11	Exon 3	c.592A > T	N198Y	1 (1Finnish)	1 (1/1)	10.1^*^	7.2^*^	23
12	Exon 3	c.593A > G	N198S	2 (1Amercian, 1British)	3 (3/8)	7.3	9.7	19
13	Exon 3	c.640G > A	A214T	1 (1Netherlandish)	7 (7/12)	/	/	24
14	Exon 3	c.641C > T	A214V	1 (1British)	0 (0/1)	/	/	11
15	Exon 4	c.724G > A	A242T	6 (3British, 1Chinese, 1French, 1Polish)	2 (2/9)	6.9	6.2	10,13,14,25
16	Exon 4	c.758C > T	T253I	4 (4Danish)	/	5.7	5.7	26
17	Exon 4	c.796C > T	R266C	1 (1British)	0 (0/1)	6.2	2.5	14
18	Exon 4	c.884A > G	M282V	1 (1Belagian)	0 (0/1)	6.2	8.2	27
19	Exon 20	c.4240C > A	R1414S	1 (1Chinese)	0 (0/2)	0.0	1.0	This study

^a^Severe phenotype: patients with headache, facial nerve palsy, vision/hearing loss or severe bone pain; proportion derived from the patients of severe phenotype with the total patients of the same genotype

*Mean T-score of BMD, / missing data

## Discussion

Through the study of pathogenic gene mutations in three patients with bone cortex thickening, mandibular enlargement and facial nerve compression, two novel pathogenic mutations in *LRP5* (c.586 T > G, c.4240C > A) were detected for the first time, which could lead to ADO I. By summarizing the reported cases with *LRP5* gain-of-function mutations, we found the common clinical manifestations of these patients were enlarged mandible, prominent torus palatinus, increased BMD or with thickened bone cortex. The severe case showed abnormally elevated BMD accompanied with headache, facial nerve or optic nerve compression related symptoms.

**Fig. 4:**
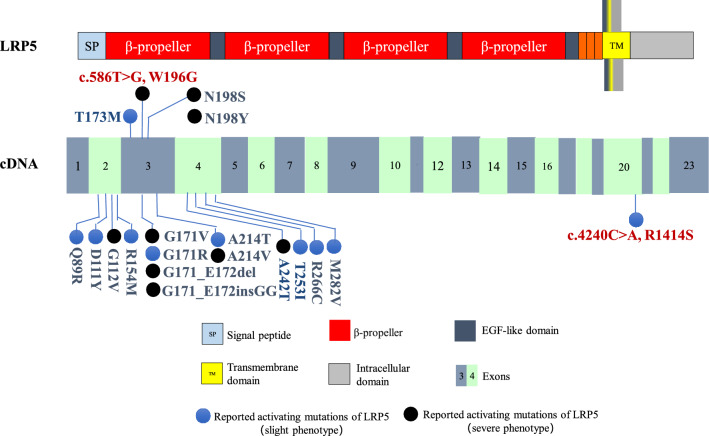
Schematic representation of the LRP5 protein, coding exons of *LRP5* cDNA, and of reported *LRP5* gain-of-function mutations. (Top) LRP5 is consisted of a large extracellular domain, a single transmembrane and an intracellular domain. The extracellular domain is comprised of a signal peptide followed by a series of four β-propeller-like structure. Exons 2–4 of *LRP5* encode the 1st β-propeller-like region, exons 20–23 encode the intracellular domain. (Bottom) A total of 19 gain-of-function mutations of *LRP5* had been reported, and mutations in exon 3 might cause severe phenotypes. The mutations in our study were indicated by *red words*

The *LRP5* gene containing 23 exons locates on chromosome 11q13, which encodes LRP5 protein, an important co-receptor of the Wnt pathway, with a large extracellular domain, a transmembrane domain and a cytoplasmic domain (Fig. 4) (He et al. 2004). LRP5 could form a receptor complex with Frizzled protein (Frz), and Wnt ligand further binds to the complex, causing phosphorylation of the cytoplasmic domain of LRP5. Then, the phosphorylated intracellular domain binds to the Axin protein, inhibiting the polymerization of Axin protein complex, leading to the release and accumulation of free β-catenin, which could be transferred into the nucleus to activate downstream target gene and increase osteoblast activity by regulating the DNA binding protein of T-cell factor/lymphoid enhancer factor (TCF/ LEF), playing an important role in regulation of bone mass (Moorer and Riddle 2018).

Activating mutations in *LRP5* can lead to increased bone mass was firstly reported in two studies in 2002 (Rickels et al. 2005; Whyte et al. 2004). Mandibular enlargement and torus palatinus prominence are the most common clinical manifestations, while the disease phenotype is heterogeneous. Mild patients may have no obvious symptoms with only bone radiographic changes or increased BMD. Severe cases may have serious bone pain, nerve compression related symptoms, and abnormally increased BMD. At present, 19 activating mutations of *LRP5* were reported, mainly distributed in exons 2–4. Common mutation types include missense mutations, insertion or deletion mutations, and hot spot mutations are c.512G > T, c.724G > A and c.758C > T. The correlation between disease genotype and phenotype still remained unclear. This study found two new mutations of *LRP5*, and the c.586 T > G (p.W196G) mutation in exon 3 caused a more serious phenotype. The phenotype caused by mutations in other exons may be lighter, more difficult to be identified and easier to be overlooked, resulting in a low detection rate of *LRP5* activating mutations.

*LRP5* gene mutation leads to changes in different domains of the receptor protein, which may be related to the disease phenotypes. *LRP5* exons 2–4 encode the 1^st^ β-propeller region of the extracellular domain of LRP5 protein, which is the direct binding site with SOST, and affects the binding of DKK-1 to the 3^rd^ β-propeller region at the same time. DKK-1 and SOST are both natural antagonists of the Wnt pathway, as a result, the extracellular domain of the LRP5 protein is essential for the activation of the Wnt signaling pathway. The amino acid changes in the 1^st^ β-propeller region of LRP5 (D111Y, G171R, A214T, A242T, T253I and M282V) could weaken its affinity with DKK-1 and SOST, leading to Wnt pathway activation and the appearance of high bone mass phenotypes (Ai et al. 2005). The *LRP5* mutation of patient 1 in this study (c.586 T > G, p.W196G) causes the 196^th^ conservative amino acid in the extracellular domain of the LRP5 protein to change from tryptophan to glycine, which may weaken its binding to DKK-1 and SOST, thereby activating the Wnt signaling pathway, and promoting bone formation, further leading to a significant increase in BMD and cranial nerve compression. The mutation (c.4240C > A, p.R1414S) of patients 2 and 3 in this study locates in exon 20 of *LRP5*, causing changes in the cytoplasmic domain of the receptor protein, which has five highly conserved PPPSPxS regions, the phosphorylation of these regions forms the binding site of Axin protein, which plays an important role in the release of downstream β-catenin (Haÿ et al. 2012). Thereby, the mutation (c.4240C > A, p.R1414S) activate the Wnt pathway, causing thickening of the bone cortex and enlargement of the mandible. Studies have confirmed that the single nucleotide polymorphism (SNP) in the cytoplasmic domain of LRP5, C.4574 T > G (p.V1525A), could enhance the binding of LRP5 with Axin protein, enhancing the differentiation of osteoblasts (Guo and Cooper 2007). By reviewing reported cases combined with this study, it is suggested that mutations in exon 3 of *LRP5* may cause severe clinical phenotypes, including G171V, G171_E172insGG, G171_E172del, W196G, N198Y, N198S, A214T, and A242T. The LRP5-SOST model constructed by Gregson et al*.* explained the possible mechanism. If the amino acid changes caused by the mutations mentioned above are in the binding site or nearby sites of the LRP5 extracellular domain with SOST, Wnt pathway will be significantly activated, causing a severe phenotype of osteosclerosis (Gregson et al. 2016). However, patients in the same family may present with different clinical phenotypes (Roetzer et al. 2018), indicating that the disease is heterogeneous.

In conclusion, LRP5 is an important co-receptor of the Wnt pathway. The activating mutations in *LRP5* can lead to torus palatinus prominence, mandibular enlargement, nerve compression, increased BMD, and thickening bone cortex. This study identified two kinds of new mutations in *LRP5* for the first time, of which the mutation in exon 3 may cause amino acid changes in the binding site of LRP5 with SOST, an antagonist protein in Wnt pathway, resulting in more severe phenotype of osteosclerosis. Moreover, the in-depth research on Wnt pathway is of great significance for studying the regulation mechanism of bone metabolism and developing new drugs for the osteoporosis treatment.

## Data Availability

The datasets for this article are not publicly available due to concerns regarding participant/patient anonymity. Requests to access the datasets should be directed to the corresponding authors. The datasets presented in this article are not readily available because no access to raw dataset of NGS is allowed other than the Beijing Genomics institution in charge of NGS. Requests to access the datasets should be directed to https://www.genomics.cn/contact.html

## References

[CR1] Ai M, Holmen SL, Van Hul W (2005). Reduced affinity to and inhibition by DKK1 form a common mechanism by which high bone mass-associated missense mutations in LRP5 affect canonical Wnt signaling. Mol Cell Biol.

[CR2] Asan Y, Xu HJ (2011). Comprehensive comparison of three commercial human whole-exome capture platforms. Genome Biol.

[CR3] Balemans W, Devogelaer JP, Cleiren E (2007). Novel LRP5 missense mutation in a patient with a high bone mass phenotype results in decreased DKK1-mediated inhibition of Wnt signaling. J Bone Miner Res.

[CR4] Balemans W, Piters E, Cleiren E (2008). The binding between sclerostin and LRP5 is altered by DKK1 and by high-bone mass LRP5 mutations. Calcif Tissue Int.

[CR5] Beals RK, McLoughlin SW, Teed RL (2001). Dominant endosteal hyperostosis. Skeletal characteristics and review of the literature. J Bone Joint Surg Am.

[CR6] Boyden LM, Mao J, Belsky J (2002). High bone density due to a mutation in LDL-receptor–related protein 5. N Engl J Med.

[CR7] Clevers H, Nusse R (2012). Wnt/β-catenin signaling and disease. Cell.

[CR8] Costantini A, Kekäläinen P, Mäkitie RE (2017). High bone mass due to novel LRP5 and AMER1 mutations. Eur J Med Genet.

[CR9] Frost M, Andersen T, Gossiel F (2011). Levels of serotonin, sclerostin, bone turnover markers as well as bone density and microarchitecture in patients with high-bone-mass phenotype due to a mutation in LRP5. J Bone Miner Res.

[CR10] Gregson CL, Wheeler L, Hardcastle SA (2016). Mutations in known monogenic high bone mass loci only explain a small proportion of high bone mass cases. J Bone Miner Res.

[CR11] Guo J, Cooper LF (2007). Influence of an LRP5 cytoplasmic SNP on Wnt signaling and osteoblastic differentiation. Bone.

[CR12] Haÿ E, Buczkowski T, Marty C (2012). Peptide-based mediated disruption of N-cadherin-LRP5/6 interaction promotes Wnt signaling and bone formation. J Bone Miner Res.

[CR13] He X, Semenov M, Tamai K (2004). LDL receptor-related proteins 5 and 6 in Wnt/beta-catenin signaling: arrows point the way. Development.

[CR14] Hernandez-Cassis C, Vogel CK, Hernandez TP (2003). Autosomal dominant hyperostosis/osteosclerosis with high serum alkaline phosphatase activity. J Clin Endocrinol Metab.

[CR15] Holdsworth G, Slocombe P, Doyle C (2012). Characterization of the interaction of sclerostin with the low density lipoprotein receptor-related protein (LRP) family of Wnt co-receptors. J Biol Chem.

[CR16] Huybrechts Y, Mortier G, Boudin E (2020). Wnt signaling and bone: lessons from skeletal dysplasias and disorders. Front Endocrinol (lausanne).

[CR17] Janer Subías E, de Arriba MA, García Iñiguez JP (2015). Autosomal dominant osteopetrosis: a presentation of 3 cases and a new gene mutation. An Pediatr (barc).

[CR18] Joiner DM, Ke J, Zhong Z (2013). LRP5 and LRP6 in development and disease. Trends Endocrinol Metab.

[CR19] Kwee ML, Balemans W, Cleiren E (2005). An autosomal dominant high bone mass phenotype in association with craniosynostosis in an extended family is caused by an LRP5 missense mutation. J Bone Miner Res.

[CR20] Little RD, Carulli JP, Del Mastro RG (2002). A mutation in the LDL receptor-related protein 5 gene results in the autosomal dominant high-bone-mass trait. Am J Hum Genet.

[CR21] Liu Y, Asan MD (2017). Gene mutation spectrum and genotype-phenotype correlation in a cohort of Chinese osteogenesis imperfecta patients revealed by targeted next generation sequencing. Osteoporos Int.

[CR22] Moorer MC, Riddle RC (2018). Regulation of osteoblast metabolism by Wnt signaling. Endocrinol Metab (seoul).

[CR23] Pangrazio A, Boudin E, Piters E (2011). Identification of the first deletion in the LRP5 gene in a patient with autosomal dominant osteopetrosis type I. Bone.

[CR24] Renton T, Odell E, Drage NA (2002). Differential diagnosis and treatment of autosomal dominant osteosclerosis of the mandible. Br J Oral Maxillofac Surg.

[CR25] Rickels MR, Zhang X, Mumm S (2005). Oropharyngeal skeletal disease accompanying high bone mass and novel LRP5 mutation. J Bone Miner Res.

[CR26] Riggs ER, Andersen EF, Cherry AM (2020). Technical standards for the interpretation and reporting of constitutional copy-number variants: a joint consensus recommendation of the American College of Medical Genetics and Genomics (ACMG) and the Clinical Genome Resource (ClinGen). Genet Med.

[CR27] Roetzer KM, Uyanik G, Brehm A (2018). Novel familial mutation of LRP5 causing high bone mass: Genetic analysis, clinical presentation, and characterization of bone matrix mineralization. Bone.

[CR28] Twells RC, Metzker ML, Brown SD (2001). The sequence and gene characterization of a 400-kb candidate region for IDDM4 on chromosome 11q13. Genomics.

[CR29] Van Wesenbeeck L, Cleiren E, Gram J (2003). Six novel missense mutations in the LDL receptor-related protein 5 (LRP5) gene in different conditions with an increased bone density. Am J Hum Genet.

[CR30] Wang C, Zhang BH, Zhang H (2013). The A242T mutation in the low-density lipoprotein receptor-related protein 5 gene in one Chinese family with osteosclerosis. Intern Med.

[CR31] Whyte MP, Reinus WH, Mumm S (2004). High-bone-mass disease and LRP5. N Engl Med.

[CR32] Williams BO (2017). LRP5: From bedside to bench to bone. Bone.

[CR33] Yu W, Qin M, Xu L (1996). Bone mineral analysis of 445 normal subjects assessed by dual X-ray absorptiometry. Chinese J Radiol.

[CR34] Yuan Q, Yang J, Sun M (2020). One family with osteosclerosis caused by D111Y mutation in the low-density lipoprotein receptor-related protein 5 gene. Chinese J Endocrinol Metab.

